# Fast Forward the Future: What Are the Key Drivers in Intelligent Sensing for Agriculture?

**DOI:** 10.3390/plants15060851

**Published:** 2026-03-10

**Authors:** Hugo Moreno

**Affiliations:** Centre for Automation and Robotics, Consejo Superior Investigaciones Científicas (CSIC), Ctra. de Campo Real, km 0.200, La Poveda, 28500 Arganda del Rey, Madrid, Spain; hugo.moreno@csic.es

## 1. Introduction

Driven by the fusion of advanced robotics and Artificial Intelligence (AI), the industry is evolving into a new era of “Agriculture 4.0”. Within the domain of AI, this shift leverages machine learning (ML) and deep learning (DL) to provide artificial perception and decision-making for UAVs, ground-based autonomous vehicles, and robotic systems, effectively redefining traditional farming through “Smart” technology. These smart farming technologies are expected to boost yields, reduce input use, and lower labor demands [1]. This Editorial aims to position the contributions of the articles featured in the Special Issue “The Future of Artificial Intelligence and Sensor Systems in Agriculture” within a broader context. The advancements highlighted in this Editorial illustrate the ongoing transition from manual, experience-based assessment to data-driven diagnostic systems. This shift is paving the way for a new generation of smart farming technologies in which ML and DL models, along with AI-powered sensor systems, play a central and transformative role. Therefore, a diverse set of innovations is presented to introduce new techniques to enable precise monitoring, automated decision-making, and robotic intervention, fundamentally transforming crop management, including weeds and pest management, disease pathology, and yield estimation.

Serving as the primary interface for data collection, sensors are fundamental components across numerous industrial sectors and modern technological systems. They provide essential data for monitoring, control, automation, and analysis in fields such as electronics, robotics, automotive engineering, healthcare, and environmental monitoring. Hence, their role is increasingly significant in agricultural and environmental data analytics [2]. In this context, this Editorial presents a diverse set of sensor-based approaches, including RGB cameras, stereo-depth systems, RVI and NDVI vegetation indices, and hyperspectral sensors, applied across multiple agricultural scenarios. These range from disease identification and pest surveillance to weed detection for spot-spraying, phenotyping, and robotic control and skill transfer.

Nonetheless, while sensors provide the raw information, it is advanced ML and DL models that fundamentally unlock the ability to interpret and operationalize these data streams. DL models, in particular, have emerged as the foundational technologies that enable this transformation, providing sophisticated methods for analyzing complex visual data from the field. At the core of modern computer vision are Convolutional Neural Networks (CNNs), which have become instrumental in image-processing tasks. CNNs automatically extract relevant features from images, such as edges, colors, and textures, through a series of convolutional and pooling layers. This hierarchical feature extraction allows the network to learn complex patterns directly from pixel data, making it highly effective for tasks like image classification and object detection in agricultural settings [3]. Another powerful architecture presented in this Editorial is transformer model-based architectures, which have recently been adapted from their origins in natural language processing to computer vision. Although CNNs are powerful at extracting local features, their restricted receptive fields limit their ability to capture global context, prompting the rise in transformer-based architectures. Furthermore, in contrast to CNNs, which depend on localized receptive fields, transformers process the entire image simultaneously, improving their ability to distinguish between weeds and crops that share similar visual traits [4]. The self-attention mechanism in transformers enables the model to effectively focus on fine image details and edges, thereby enhancing its capacity to capture long-range dependencies [5]. Due to this capability, transformers have achieved state-of-the-art results across numerous computer vision tasks, and their application in the plant disease domain has grown rapidly, including studies focused specifically on plant disease detection [6].

Furthermore, as DL models transition from high-performance servers to field operations, researchers are also focusing on “lightweighting” architectures to enable real-time inference on edge devices, mobile phones, and UAVs. To reduce energy consumption and computational costs without sacrificing accuracy, researchers are modifying the fundamental architecture of neural networks. Several specific techniques have been employed to lower parameter counts and resource consumption, such as backbone optimization, structural pruning, or dynamic routing. Regarding real-time performance on edge hardware, lightweight detection models have been developed to be implemented on mobile phones or embedded systems, i.e., edge AI devices such as Jetson Orin Nano. Finally, efficiency is not limited to inference; it also applies to the training phase of machine learning models. “Human Skill Transfer” is presented in robotic harvesting because standard reinforcement learning models (like DDPG, SAC, and TD3) often fail to converge quickly. Thus, human demonstration paths (skill transfer) are implemented into the training loop.

Accordingly, these advanced models, in combination with the presented optical sensors, establish the core architectural principles that underpin the development of more advanced and task-specific AI systems for agriculture. This Editorial illustrates three major trends: the evolution of neural network architectures to handle complex field backgrounds, the miniaturization of models for real-time mobile deployment, and the integration of human-level dexterity into robotic systems. Thus, it is presented how breakthroughs in attention-based architectures, generative adversarial networks GANs, and multimodal transformers are effectively addressing long-standing technical hurdles in weed management, pest and disease detection, phenotypic analysis, and “human-like” robotic crop harvesting, thereby bridging the gap between theoretical AI and field-ready applications.

## 2. Attention, Diffusion, and Multimodal Architectures for Disease Detection and Yield Modeling

Environmental variability, complex field backgrounds, and inconsistent real-world conditions pose significant obstacles to accurate plant disease detection [7]. Limited availability of labeled data further constrains ML and DL models’ performance [8], while background clutter and variable lighting continue to reduce detection accuracy in practical scenarios [9]. Nevertheless, the accuracy of plant disease detection has improved significantly through the adoption of attention mechanisms that mimic human visual focus to isolate pathologies from complex backgrounds. In apricot orchards, a novel Adaptive Sampling Latent Variable Network (ASLVN) integrated with a spatial state attention mechanism achieved a mean average precision (mAP) of 0.91, proving robust even in unstructured environments [10]. Similarly, state-space attention mechanisms applied to maize leaf disease have pushed precision to 0.95, outperforming standard vision transformers [11]. Improvements were also noted in wheat spike (mAP of 0.90) counting and disease detection using probability density attention mechanisms to handle dense features [12], and in radish disease detection, where hybrid (spatial and channel) attention mechanisms achieved 93% precision with a mAP of 90 [13]. On the other hand, for leafy vegetables, prototype attention mechanisms have been successfully employed to solve few-shot learning problems, i.e., to address the scarcity of agricultural disease data samples, achieving a mAP@75 of up to 0.92, even with limited training data [14]. With regard to physiological measurements, two image-based quantitative phenotyping methods were used to evaluate walnut kernels: a rule-based thresholding approach implemented with the *magick* package in R and a Mask R-CNN instance-segmentation pipeline [15]. Both methods produced highly correlated measurements, with R^2^ values above 0.98. In contrast, human scoring showed low correlation with either image-analysis method and even low agreement between evaluators themselves, underscoring the subjectivity and inconsistency inherent to manual assessments.

Beyond standard CNNs, researchers are leveraging diffusion models. A diffusion transformer combined with a knowledge graph was developed for cucumber disease detection, addressing sample imbalance to reach a (mAP) of 91%, with a frame rate of 57 frames per second (FPS) [16]. A similar diffusion transformer architecture was applied to jujube forests, achieving a F-1 score of 94% for disease detection while simultaneously estimating forest growth [17]. Nonetheless, the most notable progression is the move toward systems that process more than just images [18]. The study introduced a multimodal transformer model that integrates image, text, and sensor data. This proposed system not only detected diseases with high performance (precision, recall, and accuracy of 0.92, 0.88, and 0.91) but also generates descriptive text (image captioning), effectively functioning as an intelligent question-answering system. The study’s approach highlighted the powerful potential of multimodal data and DL for intelligent agriculture.

On the other hand, ML is also optimizing crop inputs. Because nitrogen is a key nutrient in wheat growth and yield formation, nitrogen-efficient wheat varieties were classified with 83% accuracy using SVM-XGBoost algorithms on UAV hyperspectral data [19]. The proposed method proved practical in field breeding environments and provided useful technical support to streamline the wheat breeding process. It offered an efficient and accurate way to classify nitrogen-efficient wheat varieties. Moreover, accurately predicting seed yield is crucial for enabling growers to make well-informed production decisions. In this regard, for smooth bromegrass (*Bromus inermis*), Random Forest (RF) models combined with vegetation indices identified Leaf Nitrogen Content as a critical predictor, modeling seed yield with an R2 of 0.75 [20]. Furthermore, the leaf nitrogen content proved to be a key indicator for forecasting smooth bromegrass seed yield.

## 3. Edge Computing: Lightweight Models for Real-Time Application

While accuracy is paramount, the practical adoption of AI depends on the computational efficiency on resource-constrained hardware. Computational limits, hardware constraints, and the need for lightweight models are major challenges in field deployment [21,22]. Consequently, recent research has increasingly focused on the development of computationally efficient models, concentrating on “lightweighting” models. For example, a YOLOv5s-BiPCNeXt model, optimized with MobileNeXt backbones, was implemented for eggplant disease detection. Running on a Jetson Orin Nano as an edge device, the model reached an average processing speed of 26 FPS, satisfying real-time performance requirements [23]. Similarly, a novel approach was introduced, implementing a DL model for grape disease detection that incorporates multimodal data and parallel heterogeneous activation functions [23]. Furthermore, in contrast to the previous study, the authors demonstrated even higher inference speeds by proposing a lightweight version of the model, deployed on an iPhone 15, where it achieved a real-time performance of 56 FPS. Another way to improve computational efficiency and speed is through hyperparameter optimization. Optimizing parameters such as learning rate, batch size, and weight decay helps accelerate model convergence while reducing the likelihood of overfitting or underfitting. In one study considering tomato leaf disease, extensive hyperparameter optimization of the YOLOv11m model using random search algorithms resulted in a fitness score of 0.99 [23]. Furthermore, in the domain of wild plant recognition, a lightweight Faster R-CNN utilizing a “Split SAM” lightweight spatial attention mechanism was used to improve detection accuracy without expanding the model’s architecture [24]. The model improved inference speed and accuracy on forestry devices without requiring high-performance servers.

## 4. Advance Deep Learning Models for Weeds and Pests Management

The integration of DL architectures into site-specific weed management (SSWM) represents a transformative transition regarding broadcast herbicide application or mechanical or physical control. Traditionally, weed control has relied upon uniform “blanket” spraying, a method that inevitably leads to the over-application of chemicals on bare soil and non-target crops [25]. However, the advent of neural networks has enabled a “see-and-spray” paradigm, wherein herbicides are deployed site-specifically upon the detection of specific weed species. In maize fields, a study evaluated advanced DL models to detect inter- and intra-row weeds. Among the evaluated models, YOLOv11 achieved the best overall performance, reaching a 97.5% mAP while operating in real time at 34 FPS. Hardware testing identified YOLOv11m as the most practical architecture for on-field deployment due to its strong accuracy (94.4% mAP) and lower energy demand [26]. In contrast, a lightweight weed-detection model based on a latent diffusion transformer, designed for high accuracy and real-time performance on mobile devices, was proposed [27]. By combining latent-space feature extraction, self-attention, pruning, and quantization, the model achieved strong results (precision 0.92, recall 0.89, mAP 0.91) and outperformed mainstream DL models, especially in complex agricultural images. Regarding aerial weed imagery, a study using UAV-based spot-spraying demonstrated that this technology can reduce herbicide use by 47% compared with conventional broadcast applications, without affecting crop yield or weed-control performance [28]. The automatic classifier, based on a CNN architecture (MobileNetV2), correctly identified 94% of weed plants in the UAV images. As a result, spot-spraying achieved up to 86% weed-control efficacy, matching the effectiveness of broadcast herbicide treatments. On the other hand, with respect to pest surveillance, UAVs are also revolutionizing pest control. A modified YOLOv5s model (incorporating advanced attention modules, expanded cross-stage partial network modules, and refined multiscale feature extraction mechanisms) was successfully deployed to detect specific agricultural pests, i.e., five distinct insect species, ants, grasshoppers, palm weevils, shield bugs, and wasps. The system achieved a mAP of 95.0%, demonstrating the feasibility and effectiveness of drone-based pest-monitoring ecosystems [28].

## 5. Artificial Perception and “Human-like” Manipulation

Advances in artificial perception and robotic manipulation are transforming the agricultural sector, addressing long-standing challenges associated with labor shortages, crop fragility, and the demand for higher production efficiency. While computer vision has reached considerable maturity, enabling robust detection, segmentation, and classification of crops and weeds under real-world field conditions, the integration of perception with precise physical interaction remains one of the most complex frontiers in agricultural robotics. Recent research has therefore transitioned toward incorporating demonstrations and human behavioral priors into learning algorithms, enabling robots to imitate skilled operators and refine their motor execution. To prevent damage to delicate crops, robotic systems are increasingly integrating “human skill transfer.” A robotic arm designed for tomato bunch harvesting utilized an improved Deep Deterministic Policy Gradient (DDPG) model trained on human demonstration paths, improving destination accuracy by 51.3% [29]. Efficiency in harvesting is also being addressed through multi-arm systems. A twin-arm apple-harvesting robot employing “U-tube” optimization protocol for resource distribution achieved parallel operation ratios up to 99% with zero limb interference [30]. To support these robotic eyes in the field, where motion blur is common, new lightweight Generative Adversarial Networks (GANs) can be implemented. This is the case for AGG-DeblurGAN, which has been developed to restore image quality in real-time, boosting detection by mAP by over 86% in citrus orchards [31]. Together, these developments illustrate a fundamental transformation in agricultural automation: from robots that merely perceive to robots that skillfully interact. As artificial perception continues to merge with advanced motor control, agricultural robotics is rapidly approaching the capability to execute delicate harvesting operations that were once achievable only by experienced human labor.

## 6. Conclusions

As the global population continues its upward trajectory, the agricultural sector faces a dual mandate of optimizing yields to ensure food security while simultaneously minimizing the ecological footprint through the reduction in chemical inputs and the rigorous preservation of soil health. The transition toward AI management systems is a systemic necessity dictated by the complexity of modern agricultural variables and the urgent requirement for sustainable intensification. In this context, the contemporary agricultural landscape is currently traversing a fundamental paradigm shift, transitioning from conventional methodologies toward a cutting-edge technological framework characterized by precision, automation, and data-driven decision-making. AI models continue to face challenges in accurately detecting plant diseases due to complex field conditions and the scarcity of labeled datasets, which limit the performance of traditional ML and DL models. Recent research has addressed these constraints by integrating attention mechanisms, advanced neural architectures, and multimodal learning approaches capable of combining images, text, and sensor data. Such multimodal systems not only improve disease classification but also enable caption generation and question answering, supporting more context-aware decision-making. At the same time, data-driven methods for crop phenotyping and nutrient optimization further highlight AI’s growing role in promoting sustainable agricultural production.

On the other hand, the practical deployment of AI in agriculture also depends on computational efficiency, as many systems must operate on resource-constrained hardware in the field. This has led to increased emphasis on lightweight architectures, edge-device optimization, and fast inference strategies. Research on multimodal learning with heterogeneous activation functions shows that models can maintain high accuracy while significantly reducing computational load, and hyperparameter optimization continues to enhance performance. These developments demonstrate that efficiency is a core requirement for turning AI research into usable agricultural tools, with model compression, hardware-aware design, and efficient attention mechanisms enabling high-performance detection in real-world settings. Within SSWM, DL is marking a significant evolution from uniform herbicide spraying to highly targeted interventions. Real-time neural models now provide reliable species-level weed identification in maize fields, while lightweight transformer-based models incorporating latent diffusion show improved robustness in complex imagery and can be deployed on mobile or edge devices. Aerial approaches are also advancing, with UAV-based spot-spraying systems using high-resolution weed maps to cut herbicide use nearly in half without reducing weed control or yield.

Collectively, these innovations confirm that AI-driven perception, both on the ground and from the air, is becoming essential to next-generation smart farming technologies, reducing chemical inputs, improving accuracy, and increasing operational efficiency as agriculture moves toward more sustainable and economically efficient production systems. Despite these advancements and the significant investments from both the public and private sectors, several obstacles remain. The path to a fully integrated digital farm is often hindered by a lack of industry standardization, limited interconnectivity between different technological platforms, and gaps in the availability of actionable information. These barriers must be overcome to realize the full potential of smart agriculture. We are standing at the beginning of a new era. In the decades ahead, these innovations will fundamentally transform agricultural management, shaping a system that is more efficient, more resilient, and more environmentally and economically sustainable.

## Data Availability

No data are available for this research.

## References

[1] Nettle R., Ingram J. (2025). Smart farming technologies and changes to farm work: New insights into on-farm experiences. Technol. Forecast. Soc. Change.

[2] Raj M., Prahadeeswaran M. (2025). Revolutionizing agriculture: A review of smart farming technologies for a sustainable future. Discov. Appl. Sci..

[3] Rai N., Zhang Y., Ram B.G., Schumacher L., Yellavajjala R.K., Bajwa S., Sun X. (2023). Applications of deep learning in precision weed management: A review. Comput. Electron. Agric..

[4] Singh P., Zhao B., Shi Y. (2025). Computer Vision for Site-Specific Weed Management in Precision Agriculture: A Review. Agriculture.

[5] Wang S., Zhang X., Ren L., Li J. (2026). HCT-Net: Hybrid CNN-transformer network with multi-scale feature aggregation and progressive decode for medical image segmentation. Int. J. Mach. Learn. Cybern..

[6] Elghawth R., Abbaoui W., Ariss A., Ziti S. (2025). Deep Learning for Transformer-Based Plant Disease Detection: A Bibliometric Analysis. Eng. Proc..

[7] Shafay M., Hassan T., Owais M., Hussain I., Khawaja S.G., Seneviratne L., Werghi N. (2025). Recent advances in plant disease detection: Challenges and opportunities. Plant Methods.

[8] Xu M., Yoon S., Jeong Y., Park D.S. (2022). Transfer learning for versatile plant disease recognition with limited data. Front. Plant Sci..

[9] Yu C., Xie J., Adrian Tony F.J. (2025). BGM-YOLO: An accurate and efficient detector for detecting plant disease. PLoS ONE.

[10] Han B., Duan P., Zhou C., Su X., Yang Z., Zhou S., Ji M., Xie Y., Chen J., Lv C. (2024). Implementation and Evaluation of Spatial Attention Mechanism in Apricot Disease Detection Using Adaptive Sampling Latent Variable Network. Plants.

[11] Zhu T., Yan F., Lv X., Zhao H., Wang Z., Dong K., Fu Z., Jia R., Lv C. (2024). A Deep Learning Model for Accurate Maize Disease Detection Based on State-Space Attention and Feature Fusion. Plants.

[12] Li R., Hong W., Wu R., Wang Y., Wu X., Shi Z., Xu Y., Han Z., Lv C. (2024). Enhancing Wheat Spike Counting and Disease Detection Using a Probability Density Attention Mechanism in Deep Learning Models for Precision Agriculture. Plants.

[13] Ji M., Zhou Z., Wang X., Tang W., Li Y., Wang Y., Zhou C., Lv C. (2024). Implementing Real-Time Image Processing for Radish Disease Detection Using Hybrid Attention Mechanisms. Plants.

[14] Hai T., Shao Y., Zhang X., Yuan G., Jia R., Fu Z., Wu X., Ge X., Song Y., Dong M. (2025). An Efficient Model for Leafy Vegetable Disease Detection and Segmentation Based on Few-Shot Learning Framework and Prototype Attention Mechanism. Plants.

[15] Lee S.H., McDowell S., Leslie C., McCreery K., Earles M., Brown P.J. (2025). Comparison of Mask-R-CNN and Thresholding-Based Segmentation for High-Throughput Phenotyping of Walnut Kernel Color. Plants.

[16] Li R., Su X., Zhang H., Zhang X., Yao Y., Zhou S., Zhang B., Ye M., Lv C. (2024). Integration of Diffusion Transformer and Knowledge Graph for Efficient Cucumber Disease Detection in Agriculture. Plants.

[17] Hu X., Zhang Z., Zheng L., Chen T., Peng C., Wang Y., Li R., Lv X., Yan S. (2024). Enhancing Jujube Forest Growth Estimation and Disease Detection Using a Novel Diffusion-Transformer Architecture. Plants.

[18] Lu Y., Lu X., Zheng L., Sun M., Chen S., Chen B., Wang T., Yang J., Lv C. (2024). Application of Multimodal Transformer Model in Intelligent Agricultural Disease Detection and Question-Answering Systems. Plants.

[19] Li Y., Wang C., Zhu J., Wang Q., Liu P. (2025). Classification of Nitrogen-Efficient Wheat Varieties Based on UAV Hyperspectral Remote Sensing. Plants.

[20] Ou C., Jia Z., Sun S., Liu J., Ma W., Wang J., Mi C., Mao P. (2024). Using Machine Learning Methods Combined with Vegetation Indices and Growth Indicators to Predict Seed Yield of Bromus inermis. Plants.

[21] Joshi H. (2024). Edge-AI for Agriculture: Lightweight Vision Models for Disease Detection in Resource-Limited Settings. arXiv.

[22] Min X., Ye Y., Xiong S., Chen X. (2025). Computer Vision Meets Generative Models in Agriculture: Technological Advances, Challenges and Opportunities. Appl. Sci..

[23] Xie Z., Li C., Yang Z., Zhang Z., Jiang J., Guo H. (2024). YOLOv5s-BiPCNeXt, a Lightweight Model for Detecting Disease in Eggplant Leaves. Plants.

[24] Cui Z., Chen Z., Cui X. (2025). Balancing Accuracy and Computational Efficiency: A Faster R-CNN with Foreground-Background Segmentation-Based Spatial Attention Mechanism for Wild Plant Recognition. Plants.

[25] López-Correa J.M., Moreno H., Ribeiro A., Andújar D. (2022). Intelligent Weed Management Based on Object Detection Neural Networks in Tomato Crops. Agronomy.

[26] Gómez A., Moreno H., Andújar D. (2025). Intelligent Inter- and Intra-Row Early Weed Detection in Commercial Maize Crops. Plants.

[27] Cui Y., Yang Y., Xia Y., Li Y., Feng Z., Liu S., Yuan G., Lv C. (2024). An Efficient Weed Detection Method Using Latent Diffusion Transformer for Enhanced Agricultural Image Analysis and Mobile Deployment. Plants.

[28] Allmendinger A., Spaeth M., Saile M., Peteinatos G.G., Gerhards R. (2024). Agronomic and Technical Evaluation of Herbicide Spot Spraying in Maize Based on High-Resolution Aerial Weed Maps—An On-Farm Trial. Plants.

[29] Zhang Y., Li Y., Feng Q., Sun J., Peng C., Gao L., Chen L. (2025). Compliant Motion Planning Integrating Human Skill for Robotic Arm Collecting Tomato Bunch Based on Improved DDPG. Plants.

[30] Yan B., Li X. (2025). Task-Parceling and Synchronous Retrieval Scheme for Twin-Arm Orchard Apple Tree Automaton. Plants.

[31] Huang Y., Li H., Yang Y., Li C., Wang L., Wang P. (2025). Lightweight GAN for Restoring Blurred Images to Enhance Citrus Detection. Plants.

